# AGING WITH DISABILITY AND AGING INTO DISABILITY: HOW INCOME AND MISTREATMENT EXPERIENCE ALTER SELF-PERCEIVED AGE?

**DOI:** 10.1093/geroni/igae098.3787

**Published:** 2024-12-31

**Authors:** Yalu Zhang, Ziyan Chen

**Affiliations:** Stony Brook University, Stony Brook, New York, United States; Peking University, Beijing, Beijing, China

## Abstract

Individuals who experience early-onset disability (aging with disability) might have a lifetime of adaptation and resilience that shapes their views on aging differently from those who encounter disability in later life (aging into disability). Conversely, older adults with late-onset disability might maintain higher financial stabilities which could significantly impact their aging experience. It remains understudied if these contrasting effects tend to offset each other or if one has a more significant impact. Using 2018 China Longitudinal Aging Social Survey data, this study examined whether the onset timing of disability shapes attitudes towards aging differently, and how the experience of elder abuse could alter this association. Attitudes toward aging were measured both in subjective age and an index of self-perceived capabilities and values. Consistent with previous findings, this study found that the majority of older adults with impairments in (instrumental) activities of daily living had the onset of disabilities after the age of 65 (n=660, 91%). This study uniquely identified that, compared to older adults with early-onset disabilities, those who had later-onset disabilities perceived their aging, capabilities, and social values more negatively (O.R.=2.56, p=0.003). The odds drastically increased to 9.03 times if limiting the analytical sample to those who experienced elder abuse in the last 12 months (p=0.013). This study provides valuable insights into how the timing of disability onset influences older adults’ attitudes toward aging. The marked impact of elder abuse on these perceptions highlights an urgent need for targeted prevention and interventions among those who have disabilities with aging.

